# Impact of Autism on the Relation Between Sleep and Life Satisfaction in Japanese Adults

**DOI:** 10.3390/diseases12120305

**Published:** 2024-11-28

**Authors:** Yuji Shimizu, Tomokatsu Yoshida, Keiko Ito, Kumiko Terada, Nagisa Sasaki, Eiko Honda, Kazushi Motomura

**Affiliations:** 1 Epidemiology Section, Division of Public Health, Osaka Institute of Public Health, Osaka 537-0025, Japan; tmyosida@iph.osaka.jp (T.Y.); keitou@iph.osaka.jp (K.I.); terada@iph.osaka.jp (K.T.); sasaki@iph.osaka.jp (N.S.); honda@iph.osaka.jp (E.H.); motomura@iph.osaka.jp (K.M.); 2 Department of General Medicine, Nagasaki University Graduate School of Biomedical Sciences, Nagasaki 852-8501, Japan; 3 Department of Public Health, Osaka University Graduate School of Medicine, Osaka 565-0871, Japan

**Keywords:** autism spectrum disorder, quality of life, short sleep, sleep disorder

## Abstract

Background/Objectives: Sleep disorders, such as short sleep, are common comorbidities in individuals with autism spectrum disorder (ASD). Sleep quality and duration are directly associated with quality of life (QOL). Clarifying the influence of ASD on the association between short sleep duration and life satisfaction is an efficient way to improve the QOL of patients with ASD. Methods: To clarify the influence of ASD on the association between short sleep duration and life satisfaction scale scores, we conducted a web-based cross-sectional study involving 3823 Japanese adults aged 20–64 years. Results: In all the participants, a significant inverse association was observed between short sleep duration and life satisfaction. The adjusted odds ratio (OR) and 95% confidence interval (CI) of short sleep for one standard deviation (SD), the increment of life satisfaction scale (2.5 for men and 2.4 for women), was 0.76 (0.70, 0.82). When the analyses were stratified by ASD status, a significant inverse association was observed only among participants without ASD. The corresponding ORs (95% CIs) were 0.73 (0.67, 0.80) and 1.08 (0.85, 1.39) for those with and without ASD. Patients with ASD also showed a significant interaction effect on the association between short sleep duration and life satisfaction. Conclusions: Only participants without ASD showed a significant inverse association between short sleep duration and life satisfaction. Although further investigations are necessary, these results can help clarify the mechanism underlying the association between QOL, short sleep duration, and ASD.

## 1. Introduction

Recently, adult autism spectrum disorder (ASD), which reduces the quality of life (QOL) [1], has received attention because the number of individuals who are recognized as having the disorder in adulthood is increasing [2,3]. Sleep disorders are a common comorbidity in individuals with ASD [4]. Sleep quality and duration are directly associated with QOL [5]. Short sleep duration is associated with comorbidities in ASD [6], with a significant inverse correlation between sleep duration and the severity of core ASD symptoms [6]. The prevalence of bipolar disorder was significantly higher in individuals with ASD than in non-ASD controls [7].

Increased sleep duration predicted improvements in manic and psychotic symptoms [8]. Therefore, individuals with ASD and short sleep duration may have a higher risk of bipolar disorder in the manic phase. Because individuals in the manic phase might tend to increase rather than decrease their life satisfaction scale (LSS) scores, a measurement of QOL among individuals with ASD, this scale could be positively associated with short sleep.

Depression is also positively associated with short sleep duration. Among those with short sleep duration (<8 h), increased sleep duration is associated with a lower prevalence of depression [9]. Depression is often observed in individuals with ASD [10]. As depression dramatically reduces QOL among individuals with ASD, LSS scores may be inversely associated with short sleep duration.

These studies indicate that ASD may strongly influence the association between LSS scores and short sleep duration.

Because the Japanese are reported as one of the shortest sleepers in the world [11], clarifying the factors that influence the association between LSS and short sleep among the Japanese could be clinically important for developing strategies for sleep disorders. However, no study has reported the effect of ASD status on the interaction between short sleep duration and LSS.

We hypothesized that, among individuals without ASD, the LSS would be significantly inversely associated with short sleep duration but not among those with ASD. We also hypothesized that ASD status would significantly affect the interaction between the LSS and short sleep duration. Clarifying these associations is an efficient tool for broadening the clinical perspective of sleep disorders, QOL, and ASD in the general population. Subsequently, a web-based, cross-sectional study was conducted.

## 2. Materials and Methods

### 2.1. Study Population

The methods related to the present risk survey, including sleep status, have been described previously [12]. Several individuals with ASD may have difficulty participating in social services such as annual health checkups. Face-to-face communication might be inappropriate for identifying developmental and/or psychiatric disorders in the general population because these individuals have social anxiety [13]. Because annual health checkups use face-to-face communication to acquire data from individuals, face-to-face communication prevents those with developmental and/or psychiatric disorders from participating in annual health checkups. Therefore, a web-based survey was conducted in this study. Between 27 and 30 November 2023, an email requesting informed consent to participate in the survey was sent to resident monitors in Osaka Prefecture registered with Cross Marketing Inc. (Tokyo, Japan). This study was approved by the Ethics Committee of the Osaka Institute of Public Health (project registration code: 2307-01). The target population comprised 3865 participants (1926 men and 1939 women) aged 20–64. The data on the history of developmental disorders, ASD, habitual exercise status, habitual walking status, sleep quality, sleep duration, and alcohol flush status were obtained from the survey. To avoid the influence of severe sleep disorders that reflect daily life, participants who were asleep more than 18 h per day (n = 10) and those who were asleep for less than two hours (n = 32) were excluded from the survey. The remaining 3823 participants, with a mean age of 43.7 (standard deviation [SD]: ±12.2), were enrolled in the study.

### 2.2. Definitions

#### 2.2.1. LSS

To evaluate well-being, the Japanese government (Cabinet Office) created an LSS (range: 0–10) [14]. Using this scale, we evaluated the level of life satisfaction of the enrolled population. The LSS showed essentially the same values as the report from the Japanese government (5.6 ± 2.3 for men and 6.3 ± 2.2 for women) [14] and the present study population (5.4 ± 2.5 for men and 5.7 ± 2.4 for women).

#### 2.2.2. Habitual Walking

Participants were asked, “How many times per week do you take more than 10 min walking?” Information on the number of times per week was also obtained.

#### 2.2.3. Sufficient Sleep

Sleep status was assessed as follows: “Are you getting enough rest by sleep?” Participants were asked to select their status from the following options: “satisfied”, “slightly dissatisfied”, “quite dissatisfied”, or “very dissatisfied or I couldn’t sleep at all.”

Participants who selected “satisfied” were considered to have sufficient sleep (satisfactory sleep).

#### 2.2.4. Short Sleeper and Long Sleeper

Because previous studies, including Japanese studies, defined six to eight hours as normal sleep duration [15,16], short sleepers were defined as those sleeping for less than six hours, and long sleepers were defined as those sleeping for more than eight hours.

#### 2.2.5. ASD

If participants responded “yes” to the question “Have you been diagnosed with a development disorder or ASD?” they were asked to specify their disorder as follows: attention-deficit/hyperactivity disorder, ASD, high-functioning autism, Asperger’s syndrome, unspecified developmental disorder, specific learning disorder, and other developmental disorders. Participants with ASD, high-functioning autism, Asperger’s syndrome, or an unspecified developmental disorder were categorized as having ASD. Additionally, those who scored 33 or higher on the Autism Spectrum Quotient, a self-administered instrument, were considered to have ASD [17].

### 2.3. Statistical Analysis

The ASD-specific characteristics of the study population in relation to the quartile levels of the LSS are presented as percentages, except for age, which is presented as mean ± SD. Significant differences were evaluated using analysis of variance (ANOVA) for continuous variables and the chi-square test for proportions. Logistic regression models were used to calculate the odds ratios (ORs) and 95% confidence intervals (CIs) to determine the association between short sleep duration and LSS. The ASD-specific association between short sleep duration and LSS was also evaluated. A good fit was validated using the Hosmer–Lemeshow test. Factors that influence bipolar disorder, depression, and the severity of ASD might act as mediators of the association between short sleep and LSS stratified by the status of ASD. However, factors that influence both the presence of ASD and sleep quality may also act as confounders in the analysis of short sleep and LSS. Exercise, walking, and alcohol use influence the status of ASD [18,19,20] and sleep [21,22,23], and thus, these factors could act as confounders in the present analysis. Two adjusted models were employed to determine the presence of associations between short sleep and the LSS. The first model was adjusted for sex and age, and the second, a multivariate model, was adjusted for sex, age, exercise (no, yes), walking (no, yes), long sleep duration (no, yes), sufficient sleep (no, yes), and current drinking (no, yes). For the analysis of short and satisfactory sleep durations, a multivariate model was adjusted for sex, age, exercise (no, yes), walking (no, yes), long sleep duration (no, yes), and current drinking habits (no, yes). In addition, the effect of ASD on the association between short sleep duration and life satisfaction was assessed using logistic regression models. All statistical analyses were performed using SAS for Windows (version 9.4; SAS., Cary, NC, USA). Statistical significance was set at *p* < 0.05.

## 3. Results

The participants were divided into men and women. Among the study participants, 313 (8.2%) had ASD, and 906 (23.7%) had short sleep durations.

### 3.1. Characteristics of the Study Population

Table 1 shows the ASD-specific characteristics of the study population with respect to life satisfaction. Among the participants without ASD, although the LSS score was significantly positively associated with sufficient sleep and exercise, significantly inversely associated with short sleep duration. Among those with ASD, LSS scores were significantly positively associated with sufficient sleep and exercise, whereas a mirror-J-shaped association between LSS scores and long sleep duration was observed.

### 3.2. Association Between Short Sleep and LSS

The ORs (95% CIs) of short sleep duration for the LSS among the included participants are shown in Table 2. There was a significant association between short sleep duration and life satisfaction. The sex- and age-adjusted OR (95% CI) of short sleep for a 1 SD increment on the LSS was 0.72 (0.67, 0.77). Despite further adjustment for known confounding factors, the same association was observed, with the corresponding value being 0.76 (0.70 and 0.82).

### 3.3. Association Between Short Sleep and LSS Stratified by ASD Status

Table 3 shows ASD-specific associations between short sleep duration and life satisfaction. A significant inverse association between short sleep duration and LSS scores was observed among participants without ASD but not among those with ASD. The sex- and age-adjusted ORs (CIs) of short sleep for 1 SD increment of the LSS were 0.68 (0.63, 0.74) for participants without ASD and 1.10 (0.86, 1.37) for those with ASD. Similar associations were observed after adjusting for confounding factors. The adjusted ORs (CIs) were 0.73 (0.67, 0.80) for non-ASD and 1.08 (0.85, 1.39) for ASD.

ASD status also had a significant effect on the interaction between short sleep and LSS. The *p*-values of the corresponding values were 0.014 for sex- and age-adjusted values and 0.025 for further adjustment for confounding variable-adjusted models.

### 3.4. Association Between Short Sleep and Satisfactory Sleep

Table 4 shows the association between short sleep duration and sleep satisfaction. A significant inverse association was observed only in participants without ASD. However, an inverse tendency was noted for those with ASD, although not reaching statistical significance.

## 4. Discussion

The major finding of the present study was the significant inverse association between short sleep duration and life satisfaction in participants without ASD. ASD status also revealed a significant interaction effect on the association between short sleep duration and life satisfaction.

A previous web-based study of 9305 Japanese individuals aged 20–69 who were free from mental illness found that shorter sleep duration was directly associated with worse mental QOL [5]. This is compatible with the present study, which shows a significant inverse association between short sleep duration and life satisfaction. In the present study, we found no significant association between short sleep duration and life satisfaction in those with ASD.

To our knowledge, this is the first study to reveal that shorter sleep duration does not reduce life satisfaction among individuals with ASD. However, the mechanisms underlying this association remain unclear.

Regardless of sleep duration, sleep quality is associated with QOL in the general population without mental illnesses [5]. Thus, ASD can affect sleep quality. However, in the present study, ASD status was found to have a significant interaction effect on the association between short sleep duration and life satisfaction, even after adjusting for sleep quality (sufficient sleep). Therefore, not only the quality of sleep itself but also the clinical characteristics of short sleep duration related to ASD might play an important role in the present results.

A previous retrospective study that included 100 patients with mixed bipolar or manic episodes indicated that sleep might be a useful prognostic guide for the treatment of manic episodes. This was because a longer sleep duration was observed before the improvement of manic and psychotic symptoms [8]. Therefore, sleep duration among patients in the manic phase may be shorter than that among those in the transition phase, in whom short sleep duration might not be associated with reduced life satisfaction.

Short sleep duration is associated with comorbidities in ASD [6]. As increased sleep duration predicts improvements in manic and psychotic symptoms [8], the manic status could induce short sleep among participants suffering from psychotic symptoms. Mania and autism may share a common pathophysiology. Brain-derived neurotrophic factor (BDNF) contributes to the regulation of the nervous system by activating the tyrosine kinase B receptor (TrkB). Excess BDNF is also involved in the pathogenesis of mania and autism [24]. Thus, agents that decrease BDNF-TrkB signaling may be therapeutic for these disorders. Because mania and autism might have a common pathophysiology of activating the BDNF-TrkB pathway [24] owing to the similarity in symptoms, no significant association between short sleep and the LSS was observed.

Autoimmune activity is common in patients with mania and autism. A previous case-control study reported that depressed men who developed manic symptoms during the observational period had elevated immunological activity, as evaluated by C-reactive protein levels, which were greater than in those who did not [25].

The transcription factor nuclear factor NF-kappaB (NF-κB) regulates immunological activity [26]. Dysregulation of NF-κB pathway is the pathogenesis of bipolar disorder [27]. Because stimulating NF-κB activity is observed in individuals with ASD [28,29], stimulating immunological activity may trigger a common mechanism for the development of both bipolar disorder and ASD. Therefore, short sleepers may not have decreased life satisfaction among patients with manic symptoms or ASD.

However, although the LSS showed no significant association with short sleep duration among participants with ASD (Table 1), individuals with the disorder and short sleep durations may not experience sufficient sleep. As shown in Table 4, although the statistical power did not reach significance, there was an inverse relationship between short sleep duration and satisfactory sleep. This implies that for individuals with ASD and short sleep durations, satisfactory sleep might not be a determining factor for life satisfaction. However, in the present study, a mirror-J-shaped association between long sleep duration and life satisfaction was observed in participants with ASD (Table 1). Therefore, among individuals with ASD, long sleep might have a stronger association with low levels of life satisfaction than short sleep duration. Further longitudinal investigations are necessary to clarify the influence of life satisfaction on sleep duration.

To the best of our knowledge, this is the first study to show that ASD status determines the association between short sleep duration and life satisfaction. Furthermore, for participants with ASD, short sleep duration may not be an important determinant of life satisfaction.

Individuals with mental illnesses and disorders tend to experience social anxiety [13]. Therefore, acquiring accurate data from individuals through face-to-face interviews is inappropriate. Therefore, a web-based platform was selected for this study. This is one of the strengths of the present study.

This study has several clinical implications. Short sleep duration may not influence life satisfaction in individuals with ASD. Although further investigation is necessary, improvement in sleep duration may not be an efficient strategy for improving the QOL in individuals with ASD. However, individuals with ASD and short sleep durations may not have experienced satisfactory sleep. Sleep quality may not influence life satisfaction among individuals with ASD. Although further investigations are necessary, seasonal differences may influence symptoms in patients with depression and mania [30]. As individuals with ASD may share a common pathophysiological mechanism with those with mania, the symptoms of ASD may also differ seasonally.

A significant inverse correlation between sleep duration and the severity of core ASD symptoms has been reported [6]. However, even individuals with ASD show low QOL [1], and in the present study, no significant association between short sleep and life satisfaction was observed among those with ASD. Therefore, not only short sleep duration but also the severity of ASD, which relates to short sleep duration, might not be a determinant of QOL among those with ASD. Further investigation of ASD severity is necessary to clarify these associations. Because communication skills and intelligence quotient [6] influence the severity of ASD, and the family history of alcohol use disorder [31] and autoimmune disease [32] might influence the pathogenesis of ASD, these factors may influence the association between QOL and short sleep among individuals with ASD.

Our target study participants belonged to the general population and needed to use a web survey to avoid the lack of ASD identification. Annual health checkups may be influenced by a sampling bias because of the lower ability to identify ASD. However, web surveys may also be influenced by sampling bias because they may enhance the prevalence of ASD in individuals with social anxiety [13]. Therefore, web survey data, annual health checkup data, and data from medical institutions are necessary to acquire accurate ASD prevalence data.

However, the limitations of this study warrant further investigation. First, BDNF, TrkB, and NF-κB have important roles in sleep duration in individuals with ASD; the development of neural circuitry, coordination of nerve transmission, and activating inflammation related to those factors might mediate the association between sleep duration and ASD severity of ASD. However, no corresponding data were available for this study. Further studies using data on BDNF, TrkB, and NF-κB might clarify the biochemical mechanisms underlying the clinical status of ASD. This is because the present study used self-reported data, which lacks accuracy in evaluating the status and severity of ASD. Therefore, further investigation of patients in ASD-based studies is necessary. Seasonal changes in sleep duration were not evaluated in this study. Further investigations incorporating data on seasonal influences on the symptoms are required. Because this was a cross-sectional study, a causal relationship between short sleep duration and life satisfaction levels could not be established. However, ASD is a congenital disorder, and the study population comprised adults. Thus, a significant effect of the interaction on the association between short sleep duration and life satisfaction was established. Although essentially the same associations between short sleep and satisfactory sleep were observed both with and without ASD, for those with ASD, the statistical power could not reach significance because of the small number of satisfactory sleep sessions with short sleepers (n = 9). Further investigations with larger sample sizes are warranted. In addition, unknown factors that influence both ASD status and sleep quality may have influenced the present results.

## 5. Conclusions

In conclusion, even short sleep duration was inversely associated with life satisfaction levels among participants without ASD, and no significant association was observed among participants with ASD. ASD status determined the association between life satisfaction levels and short sleep duration. Although further investigation is necessary, improvement in sleep duration may not be an efficient strategy for improving QOL in individuals with ASD. These results can help clarify the mechanism underlying the association between QOL, duration of sleep, and ASD.

## Figures and Tables

**Table 1:** Autism spectrum disorder (ASD)-specific characteristics of the study population by life satisfaction scale.

		Life Satisfaction Scale				*p*
		Quartile 1 (Low)	Quartile 2	Quartile 3	Quartile 4 (High)	
**Without ASD**						
	No. of participants	764	824	1078	844	
	Men, %	44.0	57.8	49.3	43.6	<0.001
	Age, year	46.3 ± 11.5	43.4 ± 11.6	43.7 ± 12.7	44.4 ± 12.8	0.133
	Sufficient sleep, %	12.8	20.5	24.7	41.4	<0.001
	Long Sleep, %	6.0	3.0	4.2	5.2	0.028
	Short sleep, %	33.9	25.2	20.6	15.0	<0.001
	Walking, %	45.9	39.1	49.1	55.2	<0.001
	Exercise, %	26.4	28.0	35.4	36.7	<0.001
	Drinker, %	43.6	41.5	48.1	50.2	<0.001
**With ASD**						
	No. of participants	143	69	63	38	
	Men, %	57.3	68.1	58.7	78.9	0.062
	Age, year	41.7 ± 11.3	39.9 ± 12.1	39.9 ± 11.9	36.7 ± 13.2	0.132
	Sufficient sleep, %	9.8	13.0	12.7	42.1	<0.001
	Long Sleep, %	15.4	5.8	1.6	5.3	0.006
	Short sleep, %	24.5	31.9	34.9	28.9	0.430
	Walking, %	43.4	33.3	39.7	50.0	0.349
	Exercise, %	24.5	33.3	42.9	55.3	0.001
	Drinker, %	29.4	21.7	30.2	36.8	0.401

Age: mean ± 1 standard deviation.

**Table 2:** Association between short sleep and life satisfaction scale.

	Life Satisfaction Scale				*p*	1 SD Increment of the Life Satisfaction Scale
	Quartile 1 (Low)	Quartile 2	Quartile 3	Quartile 4 (High)		
**Total**						
No. of participants, n	907	824	1078	844		
No. of short sleep, n (%)	294 (32.4)	230 (25.8)	244 (21.4)	138 (15.6)		
Model 1	Reference	0.73 (0.59, 0.90)	0.57 (0.47, 0.69)	0.38 (0.30, 0.48)	<0.001	0.72 (0.67, 0.77)
Model 2	Reference	0.72 (0.58, 0.89)	0.59 (0.48, 0.73)	0.47 (0.37, 0.60)	<0.001	0.76 (0.70, 0.82)

Model 1: adjusted for sex and age. Model 2: adjusted for sex, age, drinking, exercise, walking, long sleep duration, and sufficient sleep. SD: standard deviation.

**Table 3:** Association between short sleep and life satisfaction scale stratified by autism spectrum disorder (ASD).

	Life Satisfaction Scale				*p*	1 SD Increment of the Life Satisfaction Scale
	Quartile 1 (Low)	Quartile 2	Quartile 3	Quartile 4 (High)		
**Without ASD**						
No. of participants, n	764	824	1078	844		
No. of short sleep, n (%)	259 (33.9)	208 (25.2)	222 (20.6)	127 (15.0)		
Model 1	Reference	0.67 (0.54, 0.83)	0.51 (0.41, 0.63)	0.34 (0.27, 0.44)	<0.001	0.68 (0.63, 0.74)
Model 2	Reference	0.67 (0.54, 0.84)	0.54 (0.44, 0.68)	0.43 (0.34, 0.55)	<0.001	0.73 (0.67, 0.80)
**With ASD**						
No. of participants, n	143	69	63	38		
No. of short sleep, n (%)	35 (24.5)	22 (31.9)	22 (34.9)	11 (28.9)		
Model 1	Reference	1.51 (0.79, 2.89)	1.78 (0.92, 3.43)	1.42 (0.62, 3.25)	0.139	1.10 (0.86, 1.37)
Model 2	Reference	1.24 (0.63, 2.43)	1.42 (0.72, 2.79)	1.33 (0.54, 3.26)	0.280	1.08 (0.85, 1.39)

Model 1: adjusted for sex and age. Model 2: adjusted for sex, age, drinking, exercise, walking, long sleep duration, and sufficient sleep. SD: standard deviation.

**Table 4:** Association between short sleep and satisfactory sleep.

	Short Sleep		*p*
	(−)	(+)	
**Without autism spectrum disorder (ASD)**			
No. of participants, n	2694	816	
No. of satisfactory sleep, n (%)	801 (29.7)	79 (9.7)	
Model 1	Reference	0.25 (0.20, 0.32)	<0.001
Model 2	Reference	0.27 (0.21, 0.34)	<0.001
**With ASD**			
No. of participants, n	223	90	
No. of satisfactory sleep, n (%)	36 (16.1)	9 (10.0)	
Model 1	Reference	0.65 (0.30, 1.44)	0.289
Model 2	Reference	0.71 (0.31, 1.59)	0.400

Model 1: adjusted for sex and age. Model 2: adjusted for sex, age, drinking, exercise, walking, and long sleep duration.

## Data Availability

We cannot publicly provide individual data owing to participants’ privacy, according to the ethical guidelines in Japan. Additionally, informed consent was obtained, which did not include a provision for publicly sharing the data. Qualified researchers may apply for access to a minimal dataset by contacting Dr. Yuji Shimizu, Principal Investigator, Epidemiology Section, Division of Public Health, Osaka Institute of Public Health, Osaka, Japan, at simizu@iph.osaka.jp. Please contact the Office of Data Management at epiana@iph.osaka.jp. Data request information is available at https://www.iph.osaka.jp/s016/index.html (accessed on 3 October 2024).
